# Genetic Architecture of Abdominal Fat Deposition Revealed by a Genome-Wide Association Study in the Laying Chicken

**DOI:** 10.3390/genes15010010

**Published:** 2023-12-20

**Authors:** Jun Guo, Liang Qu, Dan Shao, Qiang Wang, Yongfeng Li, Taocun Dou, Xingguo Wang, Yuping Hu, Haibing Tong

**Affiliations:** Jiangsu Institute of Poultry Science, Yangzhou 225125, China; guojun.yz@gmail.com (J.G.);

**Keywords:** abdominal fat deposition, genomic heritability, genetic correlation, body weight gain, egg production

## Abstract

Fat has a high energy density, and excessive fatness has been recognized as a problem for egg production and the welfare of chickens. The identification of a genetic polymorphism controlling fat deposition would be helpful to select against excessive fatness in the laying hen. This study aimed to estimate genomic heritability and identify the genetic architecture of abdominal fat deposition in a population of chickens from a Dongxiang blue-shelled local breed crossbred with the White Leghorn. A genome-wide association study was conducted on abdominal fat percentage, egg production and body weights using a sample of 1534 hens genotyped with a 600 K Chicken Genotyping Array. The analysis yielded a heritability estimate of 0.19 ± 0.04 for abdominal fat percentage; 0.56 ± 0.04 for body weight at 72 weeks; 0.11 ± 0.03 for egg production; and 0.24 ± 0.04 for body weight gain. The genetic correlation of abdominal fat percentage with egg production between 60 and 72 weeks of age was −0.35 ± 0.18. This implies a potential trade-off between these two traits related to the allocation of resources. Strong positive genetic correlations were found between fat deposition and weight traits. A promising locus close to *COL12A1* on chromosome 3, associated with abdominal fat percent, was found in the present study. Another region located around *HTR2A* on chromosome 1, where allele substitution was predicted to be associated with body weight gain, accounted for 2.9% of phenotypic variance. Another region located on chromosome 1, but close to *SOX5*, was associated with egg production. These results may be used to influence the balanced genetic selection for laying hens.

## 1. Introduction

Excessive fat deposition is undesirable for egg production in laying hens because it is associated with poorer feed efficiency, increases the cost of egg production and affects the health of the hens. As a consequence, interest in controlling abdominal fat pad deposition is growing in the field of layer breeding, though sufficient abdominal fat is required to safeguard egg production, especially in the later laying period. The solution requires balancing the fat pad weight with the impact on other production traits. In a suitable breeding plan, a genetic model of the selected traits should be correctly evaluated, and parameters should be exactly described. Understanding the genetic parameters and architecture of abdominal fat deposition would be useful for layer breeding.

Selecting for a thinner abdominal fat pad would produce a faster rate of genetic gain due to its moderate heritability and large genetic variance within populations. Some studies have suggested that abdominal fat is a polygenic trait with moderate to high heritability. However, most previously reported genetic parameters were estimated using juvenile meat-type chickens [1,2,3]. There are considerable differences in gene expression profiles and metabolic patterns between the young and adult stages. A longitudinal analysis also revealed that heritability changed over time [4,5], but this study is unaware of any prior efforts to assess heritability changes in laying hens, and few genetic correlation coefficients have been reported, especially with egg production.

Advances in the development of genotyping technology have enabled genetic evaluation and selection in poultry production using a genomic relationship matrix. High-throughput association studies have revealed significant associations for economically important traits, such as egg size [6], breast muscle weight [7], ascites syndrome [8], body weight [9], feed efficiency traits [10], egg production [11] and feather pecking behavior [12]. Genome-wide association studies (GWASs) have also been performed on meat-type chickens for fat deposition [13,14,15] and body weight gain [16,17]. To the best of our knowledge, no GWASs are available for other major traits in laying chickens such as fat deposition and body weight gain during the laying period.

The aims of this study were to estimate the genetic parameters between abdominal fat deposition and performance traits using a genomic relationship matrix and to evaluate the genetic architecture of fat deposition and performance traits using GWAS. The genetic knowledge of abdominal fat deposition and performance traits will show how selection can affect fat deposition and anticipate and prevent the undesirable side effects of selection.

## 2. Materials and Methods

### 2.1. Animals

Phenotypic data were obtained from an experimental farm at the Jiangsu Institute of Poultry Science. The examined population was developed by reciprocal crosses between Dongxiang blue-shelled chickens and White Leghorn. More details about the F2 population are described by Yi et al. [6]. Zero-day-old chicks were vent-sexed, wing-banded and vaccinated for Marek’s disease on day zero. All chicks were reared in battery brooders and transferred to group cages at six weeks of age. Brooders and cages were maintained at 20 to 25 °C with artificial illumination. Pullets were then transferred to single-hen cages at 17 weeks of age. The light treatment gradually increased by 1 h per week until 16 h of light was provided. The laying mash contained 16.5% crude protein and provided 2750 kcal of metabolizable energy/kg.

### 2.2. Phenotypic Measurements

Five traits were measured, including abdominal fat weight (AFW), body weight at 28 weeks (BW28) and 72 weeks of age (BW72), and egg production (EN) between 60 weeks and 72 weeks. The body weight of live birds was measured after overnight fasting. Number of eggs produced from 60 weeks to 72 weeks was recorded daily for each bird and then summated as a total. After slaughter, abdominal fat weights were measured. 

For abdominal fat percentage and egg production, the most extreme values that varied by +3 and −3 standard deviations from the mean were removed prior to the analysis. For body weight gain, outliers are worth studying and were kept in the data set by giving them the closest value within the average ± 3 SD. All experiments were approved by the Institution of Animal Care and Use Committee in Jiangsu Institute of Poultry Science (permit number: JPIAE 2011–0005, approval date: 2 July 2013).

### 2.3. DNA Extraction, Genotyping and Quality Control

Blood samples were obtained from the brachial vein by standard venipuncture. Genomic DNA was extracted from the samples by the overnight proteinase K digestion of lysed whole blood followed by phenol/chloroform extraction. A total of 1534 hens from an F_2_ resource population were genotyped with an Affymetrix^®^ Axiom^®^ 600 K Chicken Genotyping Array (GeneSeek Inc., Lincoln, NE, USA) [18]. After the quality control procedure, 22 samples with a dish quality control less than 0.82 or single-nucleotide polymorphism (SNP) call rate less than 97% were excluded from the sample pool. Markers with a missing SNP call rate exceeding 5%, minor allele frequency less than 0.01 and Hardy–Weinberg equilibrium test *p*-value less than 1 × 10^−6^ were eliminated. All SNPs located in the sex chromosomes and those not mapped in the chicken assembly were excluded from the analysis. All autosomal SNPs from 1512 qualified samples were used for imputation implemented using the Beagle Version 4 software package based on localized haplotype clustering [19]. A total of 435,243 SNPs and 1512 birds were obtained for further analyses after filtering for imputation results using PLINK (v1.90b6.21, Shaun Purcell, https://www.cog-genomics.org/plink/, accessed on 5 January 2023) [20].

### 2.4. Genome-Wide Association Analysis

Before the association test, an independent SNP set was identified using the PLINK command --indep-pairwise 25 5 0.2 for a principal component analysis (PCA). The top five principal components were assigned as covariates in the association analyses. In the present study, the effective number of independent tests was set to 59,308, estimated by sample [21], so the genome-wide suggestive and significant *p*-values were 1.69 × 10^−5^ and 8.43 × 10^−7^, respectively. The candidate genes closest to the associated SNPs in GeneCards (http://www.genecards.org/# accessed on 6 June 2023), Ensembl (http://asia.ensembl.org, version 107, accessed on 25 May 2023) and NCBI (http://www.ncbi.nlm.nih.gov, accessed on 25 May 2023) databases were searched. The positions of interesting SNPs were obtained from Ensembl version 107 and NCBI bGalGal1.mat.broiler.GRCg7b.

A conditional analysis was also performed to identify the potential associated SNPs that might be masked in the putative region by a strong signal. Briefly, the top SNP was selected into covariates. Association analysis conditioning was then implemented based on the selected SNPs to iteratively search for the top SNPs one by one using a stepwise model selection procedure until no SNP had a conditional *p*-value that passed the significance level.

GEMMA (v0.98.5, Xiang Zhou and Matthew Stephens, https://github.com/genetics-statistics/GEMMA, accessed on 15 January 2023) was used to implement a standard linear mixed model in which a single environmental variable was explained by the SNP genotype and where relatedness was introduced by a random effect [22]. The statistical model used for the association test could be represented by the following matrix expression:

y=Wα+Xβ+Zu+ε

where ***y*** is the vector of the phenotypic values for all hens; ***W*** is the incidence matrix of fixed effects, including a column of ones, house effects and the top five PCAs; α is the vector of the corresponding coefficients, including the intercept; ***X*** is the vector of marker genotypes; **β** is the corresponding effect size of the marker; ***Z*** is the design matrices relating observations to ***u***; ***u*** is the vector of random effects with *u*~(0, GRM$\sigma_{a}^{2}$), where $\sigma_{a}^{2}$ is the genetic variance, and GRM represents the genomic relationship matrix as calculated with the GCTA package and used to replace pedigree relationship matrices [23]; and *ε* is the vector of the residual variances with *ε*~N (0, I$\sigma_{e}^{2}$), where $\sigma_{e}^{2}$ is the residual variance component. The statistical model was used as described above for a single-marker GWAS. The Wald test statistic was used as a standard to select the SNPs associated with abdominal fat and growth traits. To evaluate the systematic bias of the linear regression model, the genomic inflation factor was calculated using the ratio of the median of the observed *p*-values to that of the expected test statistics. Manhattan plots were generated with R as described by Yi et al. [6]. Quantile–quantile (QQ) plots were obtained with the gap package in R [24].

### 2.5. Heritability

In order to investigate the roles of additive genetic contributions to phenotypic variations, a restricted maximum likelihood method was applied to estimate the heritability and genetic correlation coefficients based on the genomic relationship matrix in BLUPF90 software (v1.0.1, Misztal et al., http://nce.ads.uga.edu/html/projects/programs/, accessed on 15 January 2023) [25]. The genetic variance explained by chromosomes and individual SNPs with the additive genetic effect was estimated, and the mixed linear model was used to estimate the genetic variance for each chromosome. The chicken genome was partitioned into 28 autosomes and two linkage groups. The use of the GCTA software package (v1.94.1, Yang et al., https://yanglab.westlake.edu.cn/software/gcta/#Download, accessed on 15 February 2023) allowed the pedigree relationship matrix to be replaced by the genomic relationship matrix, and the eigenvectors obtained by PCA were embedded as covariates [23]. A regression analysis was used to evaluate the relationship between the variance explained by each chromosome and its length.

### 2.6. Linkage Disequilibrium Analysis and Gene Identification

Linkage disequilibrium (LD) in target regions was visualized with Haploview 4.2 [26]. The LD blocks were defined according to the Gabriel criteria [27]. Genes that overlapped in target regions were identified with the Ensembl BioMart webtool (http://www.ensembl.org/biomart/, accessed on 6 July 2023).

### 2.7. Shared Loci Analyses

Because significant correlations were found between fat deposition and production traits with a genomic relationship matrix, the following step was aimed at revealing the overlap loci that influence both fat deposition and production traits. GWAS-PW software (v0.21, Pickrell et al., https://github.com/joepickrell/gwas-pw, accessed on 6 July 2023) was applied to identify shared loci associated with a pair of traits [28]. This program used a Bayesian method to assess the posterior probabilities of association (PPA) for four models, where one block harbored a genetic variant only associated with one trait in model 1 and model 2, another block harbored a genetic variant that was associated with both traits in model 3, and the last block harbored different genetic variants that were separately associated with each trait in model 4. In model 3, a value of PPA exceeding 0.9 was considered to jointly influence both traits at the significance level, whereas a value of PPA exceeding 0.6 was considered influential at the suggestive level.

## 3. Results

### 3.1. Description of Traits and Genetic Parameters

The statistical description of abdominal fat, egg production and growth traits is summarized in Table 1. The sample size differs among traits because of missing observations. The coefficient of variation for BW72 at 13.9% and EN at 27.9% was lower than for AFP and BWG at 44.5% and 64.13%, respectively, showing that the relative internal variability for AFP and BWG was high. The highest heritability was obtained for body weight at 72 weeks. The moderate to high heritability estimated for weight traits suggests that these traits could be significantly improved.

The phenotypic and genetic coefficient parameters are presented in Table 2. In this study, AFP showed strong genetic correlations with weight traits and moderate negative genetic correlations with egg production between 60 and 72 weeks of age. Egg production showed a low genetic correlation with weight traits. Strong and positive genetic correlations among weight traits were found.

### 3.2. SNP Association and Candidate Genes

**Figure 1:**
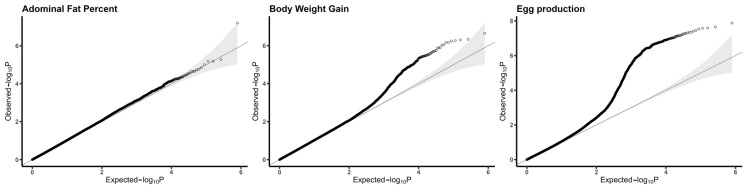
Quantile–quantile plot on the negative logarithmic scale.

The quantile–quantile (QQ) plot for each trait shows that most of the observed *p*-values fit well to the expected *p*-values, except for in the tail region of the distribution, which corresponds to associated SNPs, as shown in Figure 1. The genomic inflation factors were 0.997 for abdominal fat percent, 1.021 for egg production and 1.026 for body weight gain, indicating that inflation effects due to population stratification or an undetected genotyping error were small to negligible in each trait of interest. In the QQ plot of egg production (Figure 1), the deviation from the straight line may be due to the pleiotropy enrichment of genetic loci. Also, high-intensity selection on egg production cannot be ignored. For each trait, only the most significant regions were selected for further downstream analysis.

**Figure 2:**
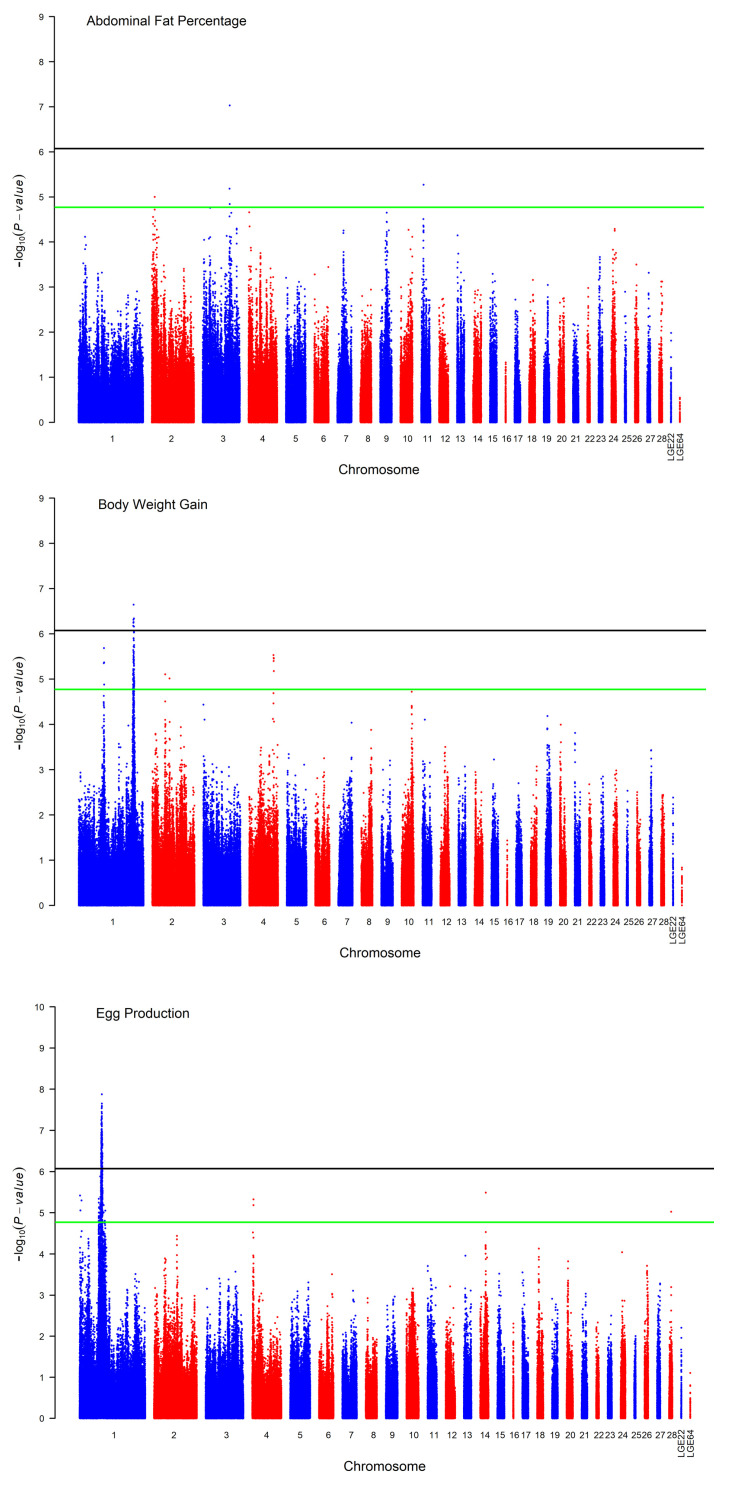
Manhattan plot of the *p*-value in the chicken GWAS. The horizontal black and green lines indicate the whole-genome significance (*p*-value = 8.43 × 10^−7^) and suggestive thresholds (*p*-value = 1.69 × 10^−5^), respectively.

One significant SNP located on chromosome 3 was rs13695700, found in the downstream region of the collagen type XII α 1 gene *COL12A1*. It had supporting SNPs spanning a region between 80.76 M and 81.06 M, as in the chicken reference genome GRCg7b, and it was identified for abdominal fat percentage based on Bonferroni correction for multiple testing, as shown in Table 3 and Figure 2. 

A total of 7 SNP effects that mapped to the long arm of chromosome 1 were identified for body weight gain, as shown in Figure 2, and 88 SNP effects reached suggestive significance. The three chromosomes *GGA1*, *2* and *4* harbored these suggestive significant SNPs. A strong association was identified for a region on GGA1 that spanned 2 Mb between 168.27 and 170.25 Mb and contained all the genome-wide and 45 suggestive significant SNPs associated with body weight gain between 28 and 72 weeks. The SNP rs317160250 was the lead SNP associated with body weight gain, as shown in Table 3. *5-Hydroxytryptamine Receptor 2A (HTR2A)* was the proximal genetic locus to the lead SNP and encoded one of the receptors as a neurotransmitter with many roles.

The GWAS for egg production revealed a significantly associated genomic region on GGA1. The SNP rs313399567 was the lead SNP located in this region associated with the number of eggs produced between 60 and 72 weeks. Due to the proximal position to the top SNP, *SRY-Box Transcription Factor 5 (SOX5)* was proposed as a candidate gene associated with egg production. The genotype and allele frequencies for this most associated SNP are given in Table 3.

- Conditional analysis

To check for any potential markers that might be masked by the single-step analysis, conditional association analyses based on significantly associated SNPs were carried out. First, any markers detected as retained after the top SNP associated with body weight gain were treated as covariates and all significant SNPs dropped out after stepwise conditional analysis. Second, the conditional analysis for egg production was repeated, and no significant association was found on chromosome 1.

- Single-chromosome heritability analysis

**Figure 3:**
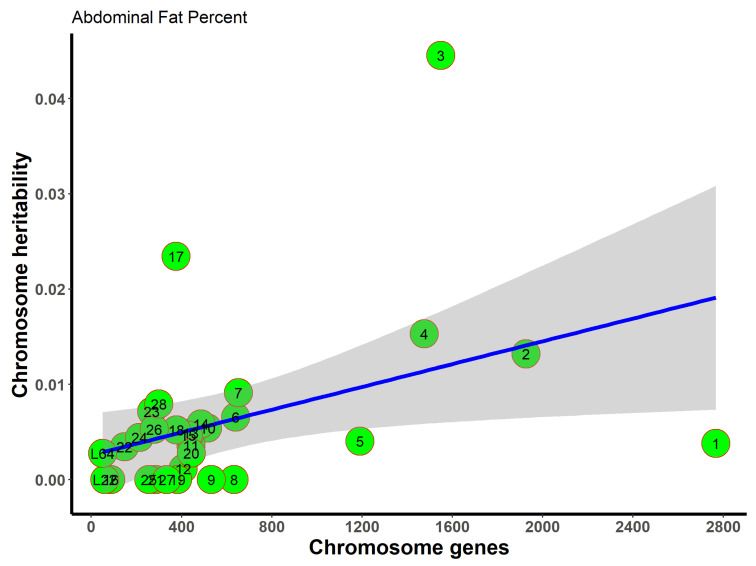
Chromosome heritability of abdominal fat percentage against the number of genes on the given chromosome. Green circles are the chromosome heritability.

To assess the distribution of the genetic components on abdominal fat percentage, we also investigated the phenotypic variance explained by each chromosome. As shown in Figure 3, the variance explained by each chromosome was proportional to its number of genes (adjusted R^2^ = 0.78). On average, more genes on a chromosome explained a larger percentage of variance. These results are consistent with the infinitesimal model theory; i.e., common variants throughout the genome accounted for the total variances. In total, 28 autosomes and two linkage groups accounted for 18.46% of the variance in abdominal fat percentage. Chromosome 3 explained 4.45% of the variance in abdominal fat percentage. These results do not differ from the univariate heritability estimates (Table 1). No convergence result was obtained on body weight gain or egg production.

- Overlap genetic locus analyses

To explore any shared loci that jointly affect fat deposition and production traits, pairwise GWASs were applied to identify which pleiotropic regions were shared between paired traits. We found that one locus reached a suggestive level for a shared association between abdominal fat deposition and body weight gain with a PPA3 of 0.85, and body weight at 72 weeks with a PPA3 of 0.81. This genomic region harbored the above-mentioned SNP mapped on chromosome 3. No region was uncovered between fat deposition and egg production.

The x-axis represents the number of genes on the chromosome, while the y-axis represents the resultant heritability in a given chromosome. The gray area around the blue line is the 95% confidence level interval predicted from the linear model. Chromosomes 3 and 17 fall outside the gray area, thereby indicating that these chromosomes could explain more heritability than expected according to the number of genes.

## 4. Discussion

### 4.1. Genetic Evaluation

The estimates of genetic correlations in this study provide unequivocal proof that abdominal fat deposition interacts with egg production and body weight gain. Unlike previous studies, the relationship matrix was constructed from genomic information rather than pedigree data. The advantage of this method lies in a more accurate description of the relative relationship, and it offers some ability to adjust for Mendelian sampling terms [29,30]. It has been observed that excessively fat hens tend to lay fewer eggs [31,32], but, to the best of our knowledge, there was no known genetic correlation with reproductive performance. Yet adequate fatness is necessary to maintain good egg production. Using the quantitative genetic method, this study found that abdominal fat deposition has a negative genetic correlation with egg production, providing a rationale to balance fat deposition and egg production during laying hen breeding programs. 

The positive genetic correlation between abdominal fat percentage and body weight gain was 0.79 ± 0.08. These results are consistent with those of a previous study by Garwood et al. [33], who reported that abdominal fat weight is positively correlated (0.84) with body weight gain. Conversely, Chen et al. [34] reported that the genetic correlation of abdominal fat proportion was weakly negatively associated with body weight gain in young broilers and unknown for the laying hen. It is generally considered that estimates of genetic correlations are less precise than those of heritability, especially for a highly variable trait such as fat deposition.

### 4.2. Genetic Architecture

In this phenotypic study of fat deposition, a large variation in abdominal fat percentage was obtained. Given the observed small genetic effect relative to the large variation in phenotype, it was not surprising that few loci were identified during the association study. The GWAS showed that one SNP located near the *COL12A1* gene was significantly associated with abdominal fat deposition. 

Adipose tissue is not merely composed of simple adipocytes, and each cell is closely associated with the extracellular matrix (ECM). *COL12A1* is a key component of ECM, which takes part in the remodeling of adipose tissue in fatness and metabolic disease [35]. In another GWAS of a broiler population, *COL12A1* was regarded as one of the candidate genes for abdominal fat deposition, and this was validated by analyzing its quantitative PCR gene expression between high- and low-fat weight groups [36]. There is also evidence that the differential expression level of *COL12A1* is related to abdominal fat deposition using RNA-seq [37] and proteomics profiles [38]. The results from this study are therefore consistent with a genetic contribution where *COL12A1* makes ECM changes leading to abdominal fat deposition. 

Knowledge of the genetic architecture of fat deposition must also be supported by the study of the interaction of related traits. These results provide essential data for a better understanding of genetic correlations related to fat deposition. The implicated region linked with *COL12A1* and its effects on the extracellular matrix remodel were also associated with phenotypic variations, such as fat deposition and body weight at 72 weeks and body weight gain. 

This study also noted with the use of GWAS that *HTR2A* was associated with body weight gain. The *HTR2A* gene encodes a serotonin receptor, and the neuroactive ligand–receptor pathway plays an important role in chicken growth by regulating food appetite [39]. Molecular genetic studies show that serotonin receptors affect appetite and body weight through the melanocortin 4 receptor [40]. Previous studies have confirmed the importance of the genomic region that includes *HTR2A* in exercising genetic control over body weight and fatness [41,42], and this study provides further evidence of an association between serotonin receptor polymorphism and body weight gain.

Another important finding was that a polymorphism in the upstream of the *SOX5* gene was significantly associated with egg production. It belongs to a member of the SOX family, which has SRY-related conserved HMG boxes in its structure. Earlier studies noted the importance of the expression of *SOX5* in influencing egg production. Through a transcriptome profile analysis, Ma et al. identified *SOX5* as one of the core genes affecting laying-rate differences [43]. Through association analyses in cattle, *SOX5* has been determined to be one of the candidate genes for female fertility [44,45], and through whole-genome scanning analyses in dairy goats, it has been noted to be related to litter size [46]. The consistency of the findings with those of other studies is encouraging; however, further validation research may be required in order to understand the effects of genetic architecture on egg production more comprehensively.

## 5. Conclusions

These results confirm the hypotheses of a potential trade-off between fatness and egg production. However, a pairwise GWAS analysis did not provide significant results for colocalization between the paired traits. There is substantial evidence that fat deposition, egg production and body weight gain are heritable and polygenic, and the majority of the genetic variance of both traits was captured by the genomic relationship matrix. A promising candidate gene involved in fat deposition was mapped to chromosome 3. A pleiotropy effect from this region appeared to be identified with pairwise GWAS. The identification of the pleiotropic region is helpful for understanding the genetic background of fatness in chickens. These results also provide additional evidence of an important role for *HTR2A* in body weight gain; a potential role for chromosomes 1, 2 and 4 in growth traits; and a key role for *SOX5* in the persistence of lay. Taken together, these results advance the understanding of the genomic architectures of fatness, body weight and egg production. 

## Figures and Tables

**Table 1:** Statistical description of abdominal fat, body weight and egg production.

Trait	Sample Size	Average ± SD	Genetic Variance	Residual Variance	Phenotypic Variance	Heritability
AFP (%)	1329	4.45 ± 1.98	0.48 ± 0.10	2.05 ± 0.10	2.53 ± 0.10	0.19 ± 0.04
BW72 (kg)	1493	1.44 ± 0.20	0.023 ± 0.003	0.018 ± 0.001	0.042 ± 0.002	0.56 ± 0.04
BWG (g)	1486	215.59 ± 138.27	4064.87 ± 751.65	12,998.17 ± 664.73	17,157.63 ± 706.11	0.24 ± 0.04
EN	1489	53.29 ± 14.89	25.53 ± 7.13	211.89 ± 9.51	237.38 ± 9.00	0.11 ± 0.03

**Table 2:** Estimates of genetic (above the diagonal) and phenotypic (below the diagonal) correlations with their approximate standard errors (in parenthesis).

Traits	AFP	BW72	BWG	EN
AFP	--	0.55 ± 0.09	0.79 ± 0.08	−0.35 ± 0.18
BW72	0.39 ± 0.03	--	0.88 ± 0.03	0.15 ± 0.14
BWG	0.42 ± 0.02	0.79 ± 0.01	--	0.14 ± 0.17
EN	−0.10 ± 0.03	0.04 ± 0.03	−0.06 ± 0.03	--

**Table 3:** Lead SNP from the significantly associated region for abdominal fat percent, body weight gain and egg production in chickens.

SNP	Chr	Position	Candidate Gene	Distance (kb)	MAF	Substitute	Proportion Phenotypic Variance	LRT *p*-Value	Length of Block (kb)	Trait
rs13695700	3	81057418	COL12A1	downstream 457 kb	0.275	G/A	0.025	9.31 × 10^−8^	6.9	Abdominal fat percent
rs317160250	1	168419997	HTR2A	upstream 164 kb	0.362	A/G	0.029	2.25 × 10^−7^	11.2	Body weight gain
rs313399567	1	65972279	SOX5	upstream 0.68 kb	0.499	A/G	0.024	7.44 × 10^−8^	299	Egg production

## Data Availability

The data presented in this study are available on request from the corresponding author. The data are not publicly available since the permission for use by the Ministry of Agriculture, the Agriculture Committee of Jiangsu Province is required.
